# Radial access for peripheral intervention: differences in comfort and care

**DOI:** 10.1186/s13019-026-04450-w

**Published:** 2026-06-19

**Authors:** Takeshi Sugimoto, Tomonori Miki, Naotoshi Wada, Shigeki Takai, Noriyuki Wakana, Hiroyuki Yamada, Kan Zen, Satoaki Matoba

**Affiliations:** 1 Department of Cardiovascular Medicine, Kyoto Tanabe Central Hospital, 6-1-6 Tanabe chuo, Kyotababe-city, Kyoto, 602-8566 Japan; 2 Department of Cardiovascular Medicine, Kyoto Prefectural University of Medicine, Kyoto, Japan; 3 Department of Cardiovascular Medicine, Kyoto Chubu Medical Center, Nantan, Japan

**Keywords:** Transradial approach, Peripheral vascular interventions, Patient satisfaction, Nurse workload, Lower extremity artery disease

## Abstract

Peripheral vascular interventions (PVIs) are less invasive alternatives to surgical bypass. However, the impact of PVIs on the post-procedural quality of life of patients and workload of nurses remains unclear. Therefore, we conducted a questionnaire-based survey including patients and nurses. The patient questionnaire consisted of eight items, including intra-procedure access site pain, post-procedure access site pain, discomfort during rest time, difficulty eating, bathroom difficulties, difficulty sleeping, difficulty walking the following day, and overall procedure satisfaction. The nurses’ workload questionnaire comprised seven items, including bathroom care, eating care, pain care, access-site care, post-procedure nurse call support, handling postoperative complications, and overall workload. Patients who underwent PVIs using the transradial approach expressed significantly less discomfort during the post-procedure rest time, difficulty eating, bathroom difficulties, difficulty sleeping, and difficulty walking the following day than those who underwent the transfemoral approach. Nurses found the post-procedural management of the transradial approach easier than that of the transfemoral approach, with significant differences in bathroom care, eating care, access-site care, and handling postoperative complications. Notably, the overall workload was significantly lower for the transradial approach than that for the transfemoral approach. Thus, the transradial arterial approach for PVI had advantages over the transfemoral approach in this study, including improved patient comfort and decreased nursing workload.

## Introduction

Recently, the incidence of lower extremity artery disease (LEAD) has markedly increased, which is largely attributed to demographic aging and the growing prevalence of lifestyle-related conditions, such as those associated with westernized diets [1, 2]. LEAD, a peripheral manifestation of systemic atherosclerosis, contributes considerably to the global disease burden by impairing patients’ daily function and, in advanced cases, can result in limb loss. These trends highlight the necessity for optimized therapeutic approaches [2].

Historically, surgical bypass was considered the standard treatment in advanced cases of LEAD. However, surgical approaches are invasive, involve prolonged recovery periods, and involve higher risks—particularly among older adult patients and those with multiple comorbidities [3, 4]. Peripheral vascular interventions (PVIs) address these limitations and are less invasive alternatives, providing comparable therapeutic outcomes with a shorter recovery period and fewer complications. PVI involves minimally invasive procedures, including angioplasty, stenting, and atherectomy [5].

The global adoption of PVI has been increasing steadily, especially in Japan, underscoring its increasing role in LEAD management [6–8]. PVI, as a minimally invasive procedure, makes it very suitable for high-risk populations, including older patients and those with notable comorbidities, for whom traditional surgical methods pose greater procedural risks [2].

PVI can be performed using various access sites, each with unique advantages and disadvantages. Traditionally, the femoral artery has been the primary access point, with the transfemoral approach offering several advantages. The femoral artery has a relatively large caliber, enabling larger sheath insertion and varying device options. Numerous studies have reported the utilization of the transfemoral approach in percutaneous coronary intervention (PCI), PVI, and structural heart procedures. The transfemoral approach remains effective; however, it is associated with an increased risk of access site complications, including hematoma, pseudoaneurysm, and substantial bleeding, particularly among patients receiving anticoagulant therapy or those with underlying coagulopathies [9, 10]. These complications may prolong hospitalization and increase healthcare expenditure, thereby offsetting procedural advantages. Advancing vascular access strategies and device technologies have therefore facilitated a paradigm shift toward radial artery access.

The radial artery approach, initially established in PCI, has demonstrated considerable reduction in access site complications [11]. Furthermore, PCI has been associated with lower in-hospital mortality rates, postprocedural bleeding, and blood transfusion in patients with chronic kidney disease, compared with the femoral artery approach [12]. This approach has recently drawn attention in PVI owing to its safety and efficacy [13]. Furthermore, endovascular therapy using the distal radial artery approach has been reported to be a less invasive alternative [14].

In PCI, the radial artery approach has consistently demonstrated superior patient satisfaction compared with the femoral artery approach. This preference is primarily driven by reduced postprocedural discomfort, expedited ambulation, and shorter recovery times, all of which contribute to a more favorable overall patient experience [15].

In aortoiliac PVI, the radial approach does not increase perioperative complications and can be performed safely while offering advantages such as shorter hemostasis time [16].

However, the impact of different PVI access sites on patients’ post-procedural quality of life and nurses’ workload remains unclear. Therefore, we conducted a post-PVI questionnaire-based survey targeting patients and nurses.

In this study, we aimed to examine how different PVI approach sites affect patients’ quality of life and nurses’ workload, thereby providing preliminary evidence regarding the potential benefits of the radial artery approach in PVI.

## Materials and methods

### Study cohort

**Fig. 1:**
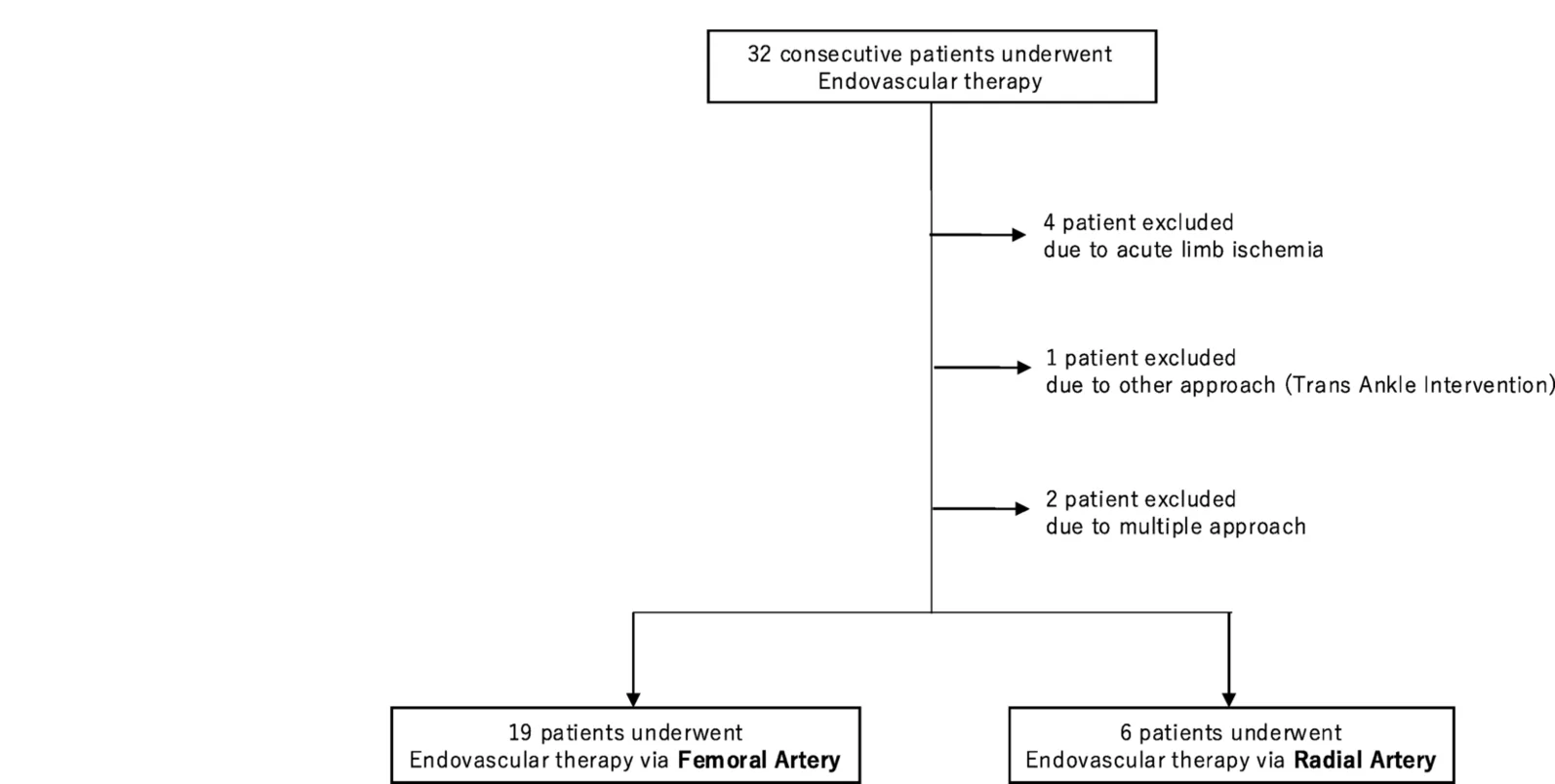
Flowchart of the study. Twenty-five patients were enrolled

The Radial Access for Peripheral Intervention: Differences in Comfort and Care study is a prospective, non-interventional, single-center clinical investigation. This questionnaire-based study was conducted between July and September 2024, involving patients and nurses following PVI. Figure 1 shows the study flowchart. The inclusion criteria were (1) age ≥ 20 years, (2) presence of ischemic symptoms caused by atherosclerotic peripheral arterial disease, and (3) having undergone PVI.

The exclusion criteria were (1) presence of an ischemic symptom following acute lower limb ischemia, (2) having undergone another approach (trans-ankle intervention), or (3) procedures requiring multiple vascular access sites. Distal puncture procedures performed without sheath insertion were not considered an additional access site because they did not affect post-procedural ambulation or bed-rest requirements and were therefore included in the analysis.

Thirty-two consecutive patients were enrolled in this study. The following patients were excluded: four patients with acute lower limb ischemia, one who underwent another approach, and two who had undergone multiple approaches. Finally, 25 patients completed the questionnaire survey (Fig. 1). This study was designed as an exploratory pilot investigation, and a formal sample size calculation was not performed. Consecutive eligible patients during the study period were prospectively enrolled.

Nineteen patients underwent endovascular therapy via the femoral approach, whereas six patients underwent therapy via the radial artery. We administered the questionnaire either during hospitalization on the day after PVI or during the waiting period before the first outpatient follow-up visit after PVI.

Furthermore, we administered surveys to both nurses responsible for daytime and nighttime shifts for the 25 patients who underwent PVI. The surveys were completed on the day following each procedure. In total, 50 nurses participated in the questionnaire, of whom 25 each were assigned to daytime and nighttime shifts. The Ethics Committee of Kyoto Tanabe Central Hospital approved this study (approval number: 2024-003, approval date: 2024/5/16). This was a prospective, non-interventional observational study. The Ethics Committee of Kyoto Tanabe Central Hospital determined that written informed consent was not required owing to the minimal-risk nature of the study. Informed consent was obtained through an opt-out process, in which the study information was publicly disclosed, and participants were given the opportunity to decline participation. The ethics committee reviewed the progress report of this study and confirmed that all methods were performed in accordance with the relevant guidelines and regulations Table 1.

**Table 1:** Characteristics of paitents

	Femoral	Radial	P
	n=19	n=6	
Age(years)	77.3±6.9	76.8±10.5	0.34
Male sex	17(85%)	3(50%)	0.07
Height	163.1±9.5	159.1±10.1	0.38
Weight	55.6±11.2	55.2±11.9	0.94
Body mass index (kg/m^2^)	20.8±3.1	21.6±3.2	0.56
Ambulatory status	18(95%)	5(83%)	0.43
Smoking history	7(37%)	3(50%)	0.65
Diabetes mellitus	13(68%)	2(33%)	0.18
Hypertension	18(95%)	6(100%)	1.00
Dyslipidemia	15(79%)	6(100%)	0.54
Renal function			
Chronic renal failure	12(63%)	2(33%)	0.35
On dialysis	2(11%)	0(0%)	1.00
Coronary artery disease	9(47%)	3(50%)	1.00
Cerebrovascular disease	6(32%)	1(17%)	0.64
Rutherford classification			
1	2(11%)	1(17%)	1.00
2	0(0%)	2(33%)	0.05
3	5(26%)	1(17%)	1.00
4	2(11%)	2(33%)	0.23
5	8(42%)	0(0%)	0.13
6	2(11%)	0(0%)	1.00
Oral administration			
Aspirin use	18(95%)	6(100%)	1.00
P2Y12 inhibitor use	18(95%)	6(100%)	1.00
Cilostazol use	1(5%)	0(0%)	1.00
Anticoagulant use	1(5%)	0(0%)	1.00
Stain use	12(63%)	5(83%)	0.62

### Items in the questionnaire sheet

**Fig. 2:**
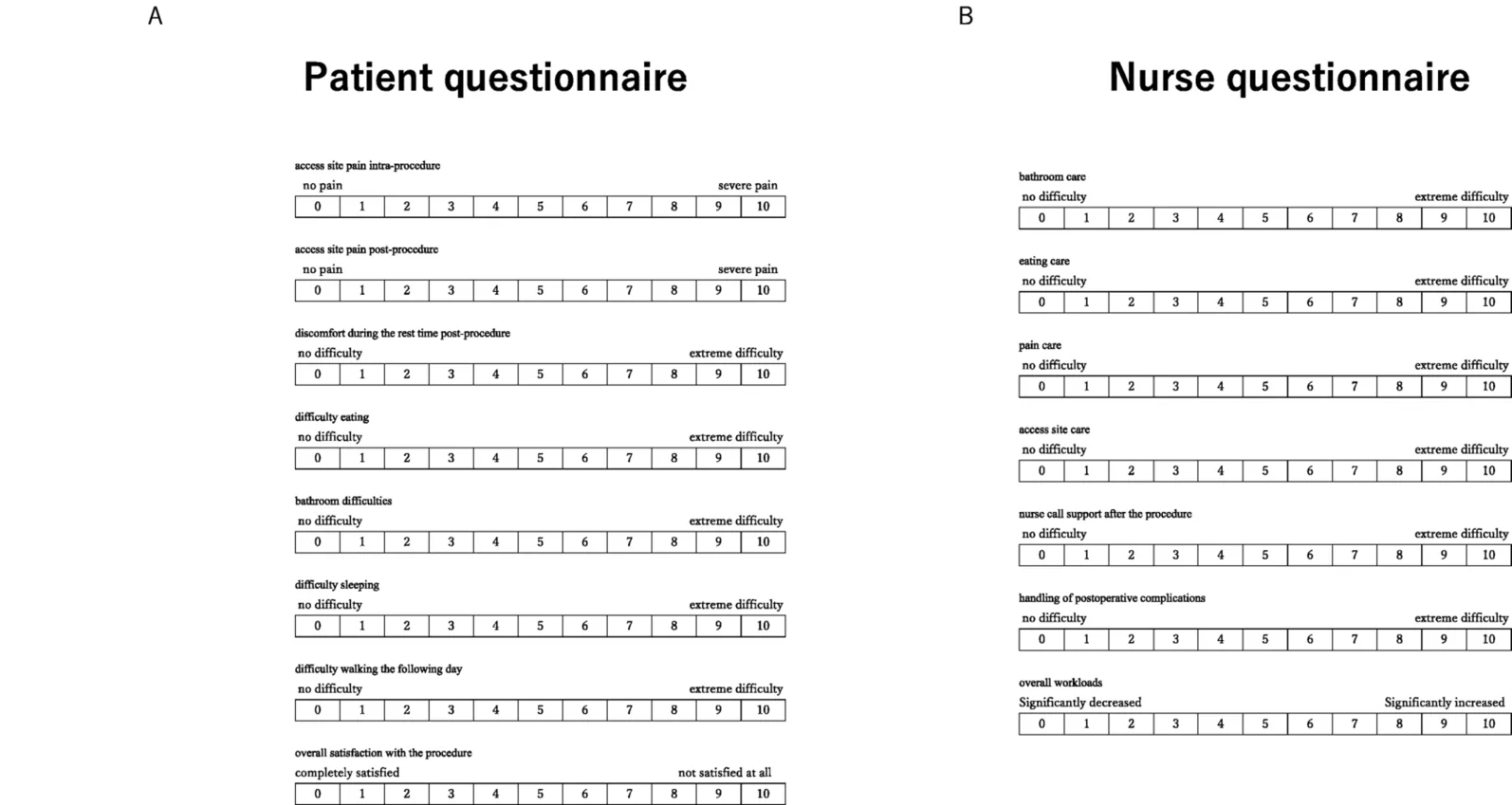
Questionnaire sheets included in the study. **a**. Patient questionnaire sheet. **b**. Nurse questionnaire sheet

The patient questionnaire sheet included eight items, including intra-procedure access site pain, post-procedure access site pain, discomfort during the rest time after the procedure, difficulty eating, bathroom difficulties, difficulty sleeping, difficulty walking the following day, and overall satisfaction with the procedure. The nurse workload questionnaire included seven items: bathroom care, eating care, pain care, access-site care, nurse call support after the procedure, handling of postoperative complications, and overall workload. An 11-point numerical rating scale (0–10) was employed to assess all items, where 0 represented the optimal condition (e.g., absence of pain or difficulty) and 10 denoted the most severe condition (e.g., intense pain or extreme difficulty) (Fig. 2). The questionnaires were pilot-tested in a small number of patients prior to initiation of the present study; however, formal validation was not performed.

### Statistical analysis

Statistical analyses were performed using GraphPad Prism software (GraphPad Software, San Diego, CA, USA). We expressed the continuous variables obtained from the questionnaire responses as mean ± standard deviation. Comparisons between the radial and femoral access groups were conducted using Welch’s t-test for all questionnaire items owing to potential variance heterogeneity and unequal sample sizes between the groups. Statistical significance was set at *p* < 0.05.

## Results

### Baseline characteristics

The femoral access group included 19 patients with a mean age of 77.3 ± 6.9 years, of whom 85% were male. The radial access group included six patients with a mean age of 76.8 ± 10.5 years, of whom 50% were male. There were no significant differences in height (163.1 ± 9.5 cm vs. 159.1 ± 10.1 cm, *p* = 0.38), weight (55.6 ± 11.2 kg vs. 55.2 ± 11.9 kg, *p* = 0.94), or body mass index (20.8 ± 3.1 vs. 21.6 ± 3.2, *p* = 0.56) between the groups.

The majority of patients in both groups were ambulatory (95% and 83% in the femoral and radial groups; *p* = 0.43). There were no significant differences in the prevalence of diabetes mellitus, hypertension, dyslipidemia, chronic renal failure, or cardiovascular comorbidities between the two groups.

Compared with patients in the radial access group, those in the femoral access group had a higher proportion of critical limb-threatening ischemia, with 42% classified as Rutherford category 5 and 11% classified as category 6. No patient in the radial access group fell in these categories (Categories 5 and 6, *p* = 0.13 and 1.00, respectively).

There were no significant differences in oral medication use between the two groups, including aspirin (95% vs. 100%, *p* = 1.00), P2Y12 inhibitors (95% vs. 100%, *p* = 1.00), statins (63% vs. 83%, *p* = 0.62), cilostazol (5% vs. 0%, *p* = 1.00), and anticoagulants (5% vs. 0%, *p* = 1.00).

The clinical success rates for the transradial and femoral approaches were 100%. No complications required blood transfusion or percutaneous hemostasis at either puncture site. However, there was one case of urethral injury during urethral balloon insertion in the femoral approach group and another case that required additional compression due to slight hematoma expansion.

### Patient questionnaire results

We excluded two patients who were unable to understand the questionnaire because of dementia. We obtained 23 (92%) valid responses, including those from 19 patients who underwent the femoral approach and four who underwent the radial approach.

**Fig. 3:**
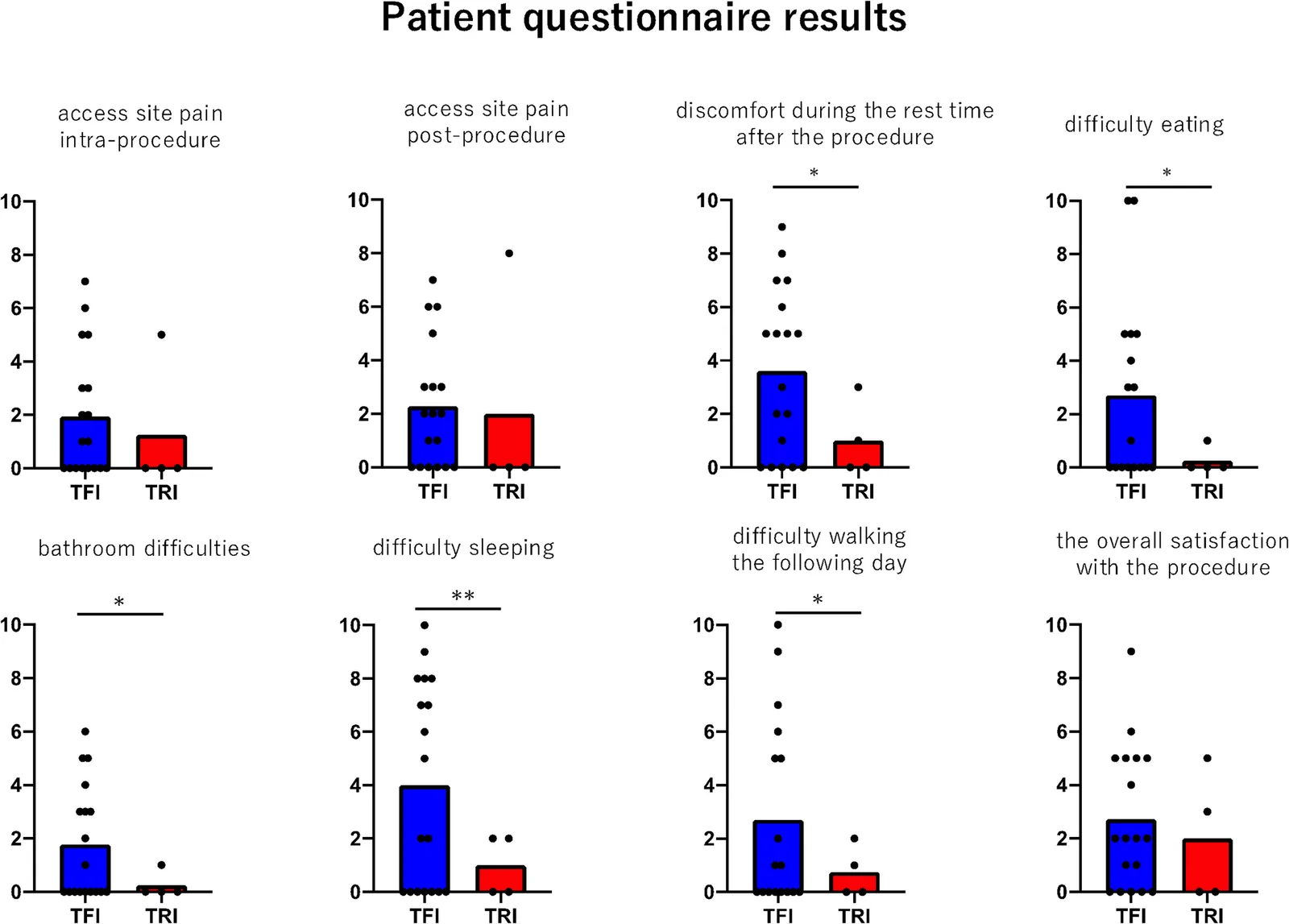
Patient questionnaire results in the study

As shown in Fig. 3, we found no significant difference in the access site pain during (1.18 ± 2.34 vs. 1.25 ± 2.5, *p* = 0.68) and after (2.53 ± 2.52 vs. 2.00 ± 4.0, *p* = 0.73) the procedure between the two groups. Discomfort during the rest time (3.74 ± 2.52 vs. 1.00 ± 1.414, *p* = 0.020), difficulty with eating (2.56 ± 3.37 vs. 0.25 ± 0.5, *p* = 0.012), bathroom difficulties (1.68 ± 2.11 vs. 0.25 ± 0.5, *p* = 0.016), difficulty sleeping (4.21 ± 3.85 vs. 1.00 ± 1.16, *p* = 0.0072), and difficulty walking the following day (3.00 ± 3.63 vs. 0.75 ± 0.96, *p* = 0.033) were lower for patients in the radial approach group than for those in the femoral approach group. Nevertheless, there was no difference in the overall procedure satisfaction between the two groups (2.73 ± 2.56 vs. 2.00 ± 2.45, *p* = 0.61).

### Nurse questionnaire results

**Fig. 4:**
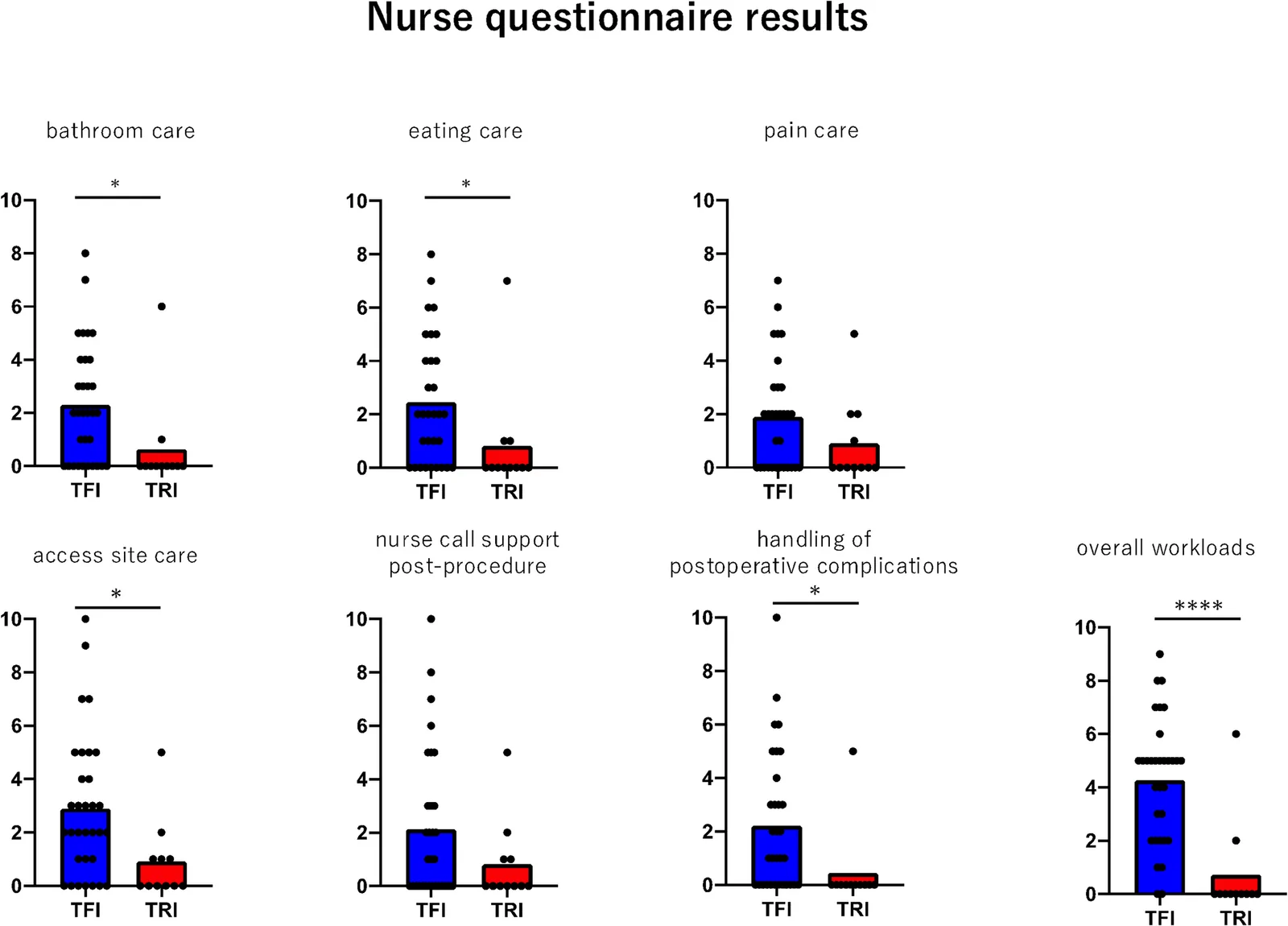
Nurse questionnaire results in the study

Of the 50 nurses who were invited to participate in the questionnaire survey, 43 completed and returned the questionnaire, yielding a response rate of 86% (22 day-shift nurses and 21 night-shift nurses). The nurses’ questionnaires are summarized in Fig. 4. The general perception of nurses was that the patients who underwent the transradial approach were more easily managed compared with those who had undergone the transfemoral approach, across all assessed aspects.

Significant differences were observed between the two approaches regarding bathroom care (2.31 ± 2.22 vs. 0.64 ± 1.80, *p* = 0.012), eating care (2.59 ± 2.46 vs. 0.82 ± 2.09, *p* = 0.031), care of the access site (2.91 ± 2.64 vs. 0.91 ± 1.51, *p* = 0.0046), and handling of postoperative complications (2.22 ± 2.59 vs. 0.45 ± 1.51, *p* = 0.010), with lower scores obtained for the transradial approach. There was a significant difference in the overall workload between the transfemoral and transradial approaches (4.28 ± 2.33 vs. 0.72 ± 1.85, respectively, *p* < 0.0001).

## Discussion

To our knowledge, this study is the first to demonstrate that different PVI access sites have an impact on patients’ post-procedural quality of life and nurses’ workload. This carries important implications for the clinical planning of PVI in patients with LEAD.

Given the rising prevalence of LEAD and the aging population, the demand for PVI procedures is expected to increase substantially. Consequently, optimizing patient comfort and minimizing nursing burden are critical considerations in procedural planning. PVI is most commonly performed via the femoral artery. However, this approach is associated with a higher incidence of vascular complications, necessitating extended postprocedural bed rest to mitigate these risks. Prolonged immobility, in turn, can lead to adverse effects such as back pain, urinary retention, and constipation. Extended bed rest also contributes to increased nursing workload, including tasks such as assisting with toileting, feeding patients unable to sit upright, and responding to frequent nurse call requests. In response to these challenges, innovations aimed at facilitating radial access for peripheral interventions have been introduced. Terumo introduced the radial-to-peripheral approach in 2019, which offers radial-specific, longer-length devices, including wires, sheaths, and stents. These advancements are expected to support broader adoption of the radial approach in PVI, potentially improving patient outcomes and reducing nursing demands.

Presently, only a few devices are compatible with radial artery access. Stents with effective working lengths reaching 200 cm are available, whereas the maximum length of drug-coated balloons is 150 cm. Guiding sheaths are offered in various lengths; however, device selection remains constrained. Recent advancements have led to the introduction of longer and more slender devices, including balloons and stent delivery systems, which may expand the feasibility of radial access.

Treatment of aortic and iliac artery lesions has increased in recent years, with the radial artery approach accounting for 16.4% of cases in Japan. In contrast, although interventions targeting the femoral and popliteal arteries have also risen, the adoption of radial access remains low, at only 2.1% nationally [ 17 ].

Future device development and varying techniques will ensure the treatment of femoral and popliteal artery lesions, as well as other arteries. Previous studies have suggested that computed tomography imaging, which is less invasive and more accessible, may serve as a useful modality for assessing the radial artery distance to the target lesion [18]. In addition, it may be possible to treat both the superficial femoral artery and below-knee at a low cost [19].

From the operator’s perspective, the transfemoral approach may offer technical advantages, including improved device support and deliverability. In contrast, transradial access may provide procedural advantages after intervention, particularly with respect to hemostasis management, which is generally simpler and less invasive than femoral artery hemostasis. Nevertheless, patients undergoing transfemoral access are generally required to remain on bed rest for at least 6 h after the procedure. In contrast, radial arterial access enables early mobilization and may improve patient comfort. In this study, the radial approach was associated with improvements across multiple quality-of-life measures.

In addition, the transfemoral group included a higher proportion of patients with advanced disease severity, including CLTI. Therefore, some of the observed differences in patient comfort and nursing workload may have been influenced by baseline clinical characteristics rather than the access site itself.

This study demonstrated that the radial approach minimized issues such as difficulty using the toilet, discomfort from the urinary balloon insertion, difficulty eating due to inability to sit up, difficulty falling asleep due to difficulty turning over, and difficulty walking the next day due to prolonged periods of rest.

The radial artery approach allows patients to sit and walk immediately, making nurses’ assistance with toileting and feeding easy. Radial artery hemostasis is often achieved using radial compression devices. Femoral puncture requires several steps, such as pulling down the patient’s pants to confirm the puncture site; however, radial puncture enables easy observation. The radial artery is more easily managed in various respects, and these factors have presumably contributed to improvements in the overall workload.

These findings suggest that the wider adoption of transradial access in PVI may not only enhance patient outcomes but also contribute to improved healthcare efficiency in an aging society. However, the present findings should be interpreted with caution because the sample size, particularly in the radial access group, was very small. Therefore, this study should be considered exploratory and hypothesis-generating, and larger multicenter studies are warranted to confirm these findings.

### Study limitations

This study has some limitations. First, this was a single-center prospective observational study, which may limit the generalizability of our findings. Second, the sample size was very small, particularly in the radial access group, where only four patients completed the questionnaire, potentially affecting statistical power. Therefore, the findings should be interpreted with caution and considered exploratory. Third, the study relied on a questionnaire-based survey, with some participants not completing their survey, introducing a possible response bias. Although the questionnaires were pilot-tested before study initiation, they were not formally validated, which may have affected the reliability of the questionnaire-based assessments. Furthermore, questionnaires were collected either during hospitalization or at the first outpatient follow-up visit. Because the timing of questionnaire completion was not systematically recorded, the possibility of recall bias cannot be excluded. Objective outcome measures, such as time to ambulation, length of hospital stay, and time to hemostasis, were not systematically evaluated and should be included in future studies. Fourth, propensity score matching was not performed, which may have led to residual confounding factors. Fifth, the PVI procedure start and end times varied among the cases, potentially contributing to inconsistencies in nursing workload.

Future studies should be conducted in a multicenter setting with larger sample sizes and propensity score matching to validate and strengthen our findings.

## Conclusions

Our study findings suggest that transradial access may improve patients’ quality of life by reducing discomfort involving rest, difficulty eating, bathroom difficulty, difficulty sleeping, and difficulty walking the day after the procedure.

Furthermore, transradial access was associated with reduced nurses’ workload, including bathroom care, eating care, care of the access site, and handling postoperative complications in patients undergoing PVI for LEAD. The overall nursing workload may be reduced by transradial access. These findings should be considered preliminary and hypothesis-generating.

## Data Availability

All data generated or analyzed during this study are included in this published article and its supplementary information files.
